# Supplementary material for: Differentially Expressed Conserved Plant Vegetative Phase-Change-Related microRNAs in Mature and Rejuvenated Silver Birch In Vitro Propagated Tissues
Source: Plants (Basel). 2023 May 16;12(10):1993. doi: 10.3390/plants12101993 (PMC10220576; doi:10.3390/plants12101993)
Supplement: Supplementary file 1 [file plants-12-01993-s001.zip › Supplementary_files_Krivmane_plants/Supplementary_file7.pdf]

Supplementary file 7. Photographs representative of the analyzed *in vitro* samples. Samples were maintained in *in vitro* culture for approximately 10 months prior to collection for analysis.

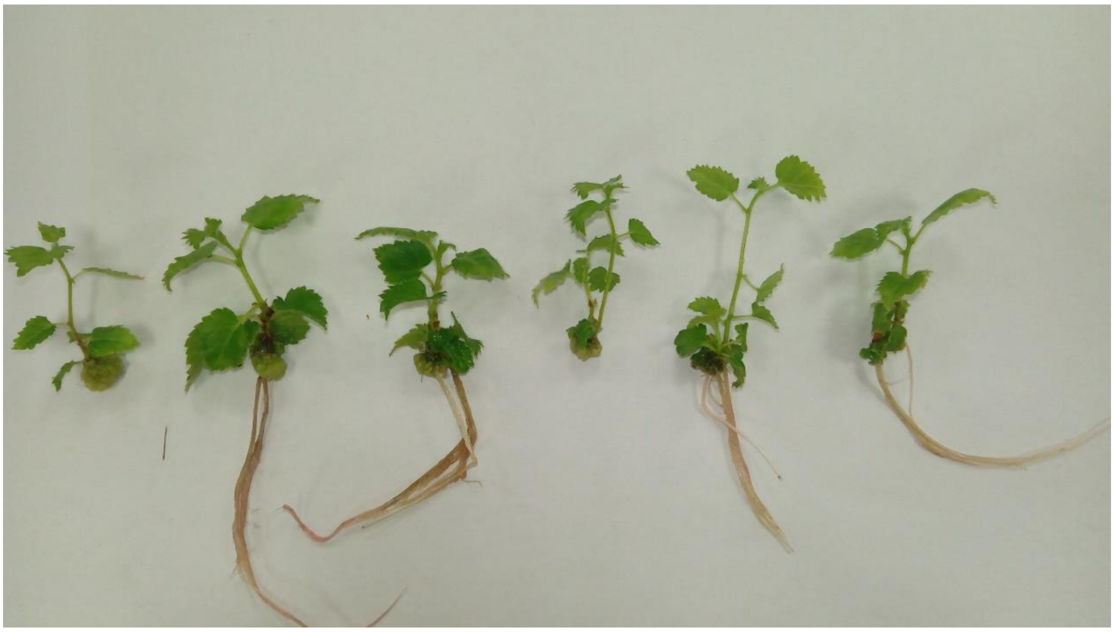

Typical rejuvenated *in vitro* shoots (REV samples).

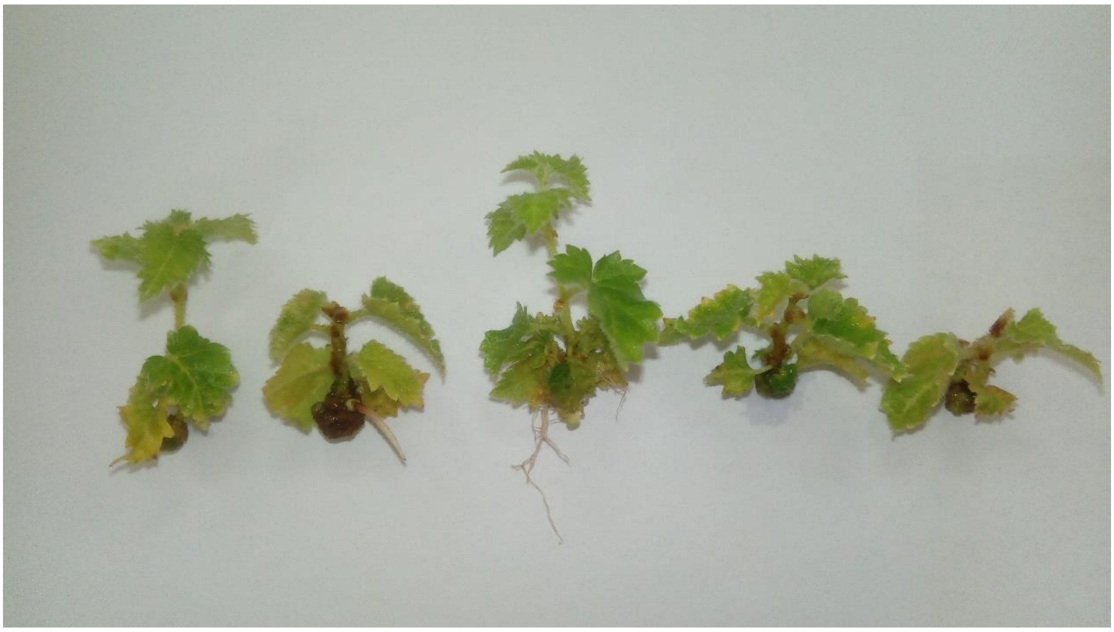

Typical mature *in vitro* shoots (IVM samples).
